# Supplementary material for: The vaginal microbiota associates with the regression of untreated cervical intraepithelial neoplasia 2 lesions
Source: Nat Commun. 2020 Apr 24;11:1999. doi: 10.1038/s41467-020-15856-y (PMC7181700; doi:10.1038/s41467-020-15856-y)
Supplement: Supplementary file 1 — Supplementary Information [file 41467_2020_15856_MOESM1_ESM.pdf]

Mitra et al.

The role of the vaginal microbiota in the regression of untreated CIN2 lesions

Supplementary Information

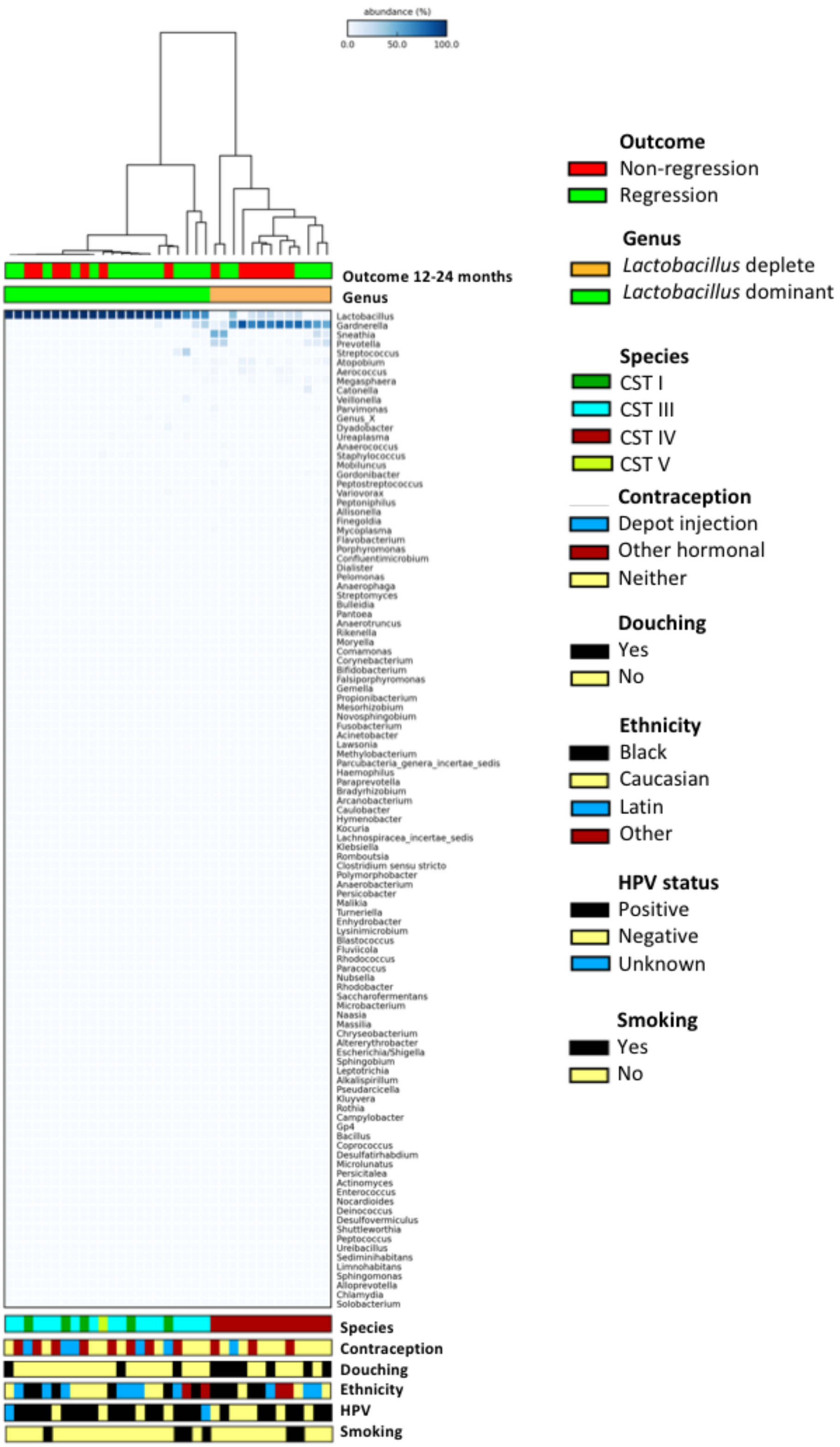

Supplementary Figure 1. Heatmap of women with persistent disease at 12 month follow-up who were followed up until 24 months, n=35.

Vaginal microbiota composition at the 12 month visit was taken as the new baseline and was classified as either *Lactobacillus spp.* dominant or deplete at genus level. There was a trend towards increased rates of regression with a *Lactobacillus spp.* Dominant VMB, however this was not statistically significant (p=0.2882, n=35).

KEY - CST: Community state type, CST I: Lactobacillus crispatus-dominant, CST III: Lactobacillus iners-dominant), CST IV: Lactobacillus spp. deplete, high diversity, VMB: vaginal microbiota

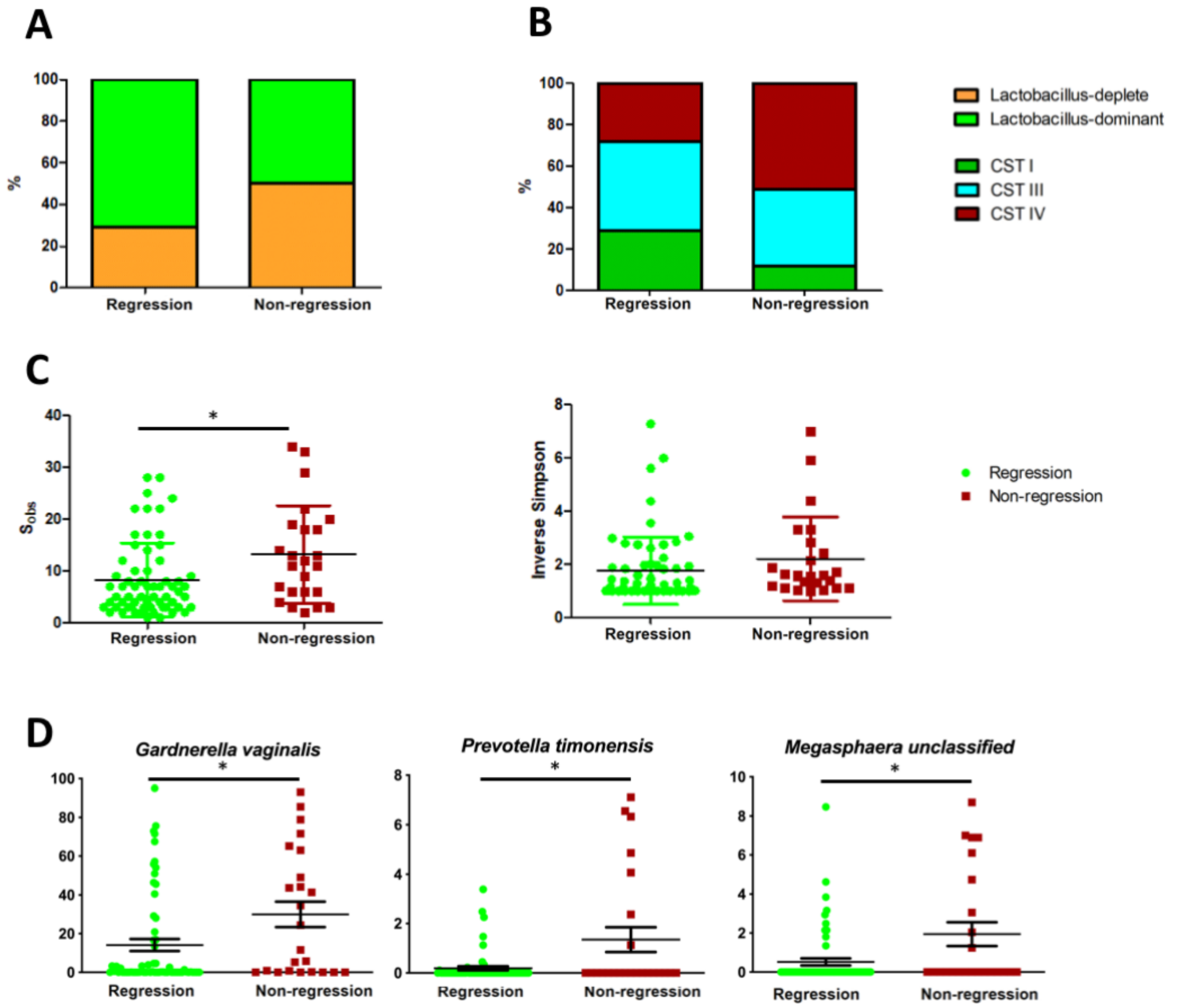

Supplementary Figure 2. Outcomes at 24 month follow-up according to baseline VMB composition at baseline
There was a trend for *Lactobacillus* spp. deplete VMB (A) and CST IV (B) were associated with lower rates of regression compared to non-regression at 24 month follow-up however this was not statistically significant. Significantly higher species richness (species observed) was seen in women who had not regressed at 12 months follow-up (p=0.0105), with a non-significant trend towards increased diversity (Inverse Simpson index) (C). The baseline vaginal microbiota of women who did not regress at 24 months was characterised by an increased abundance of *Prevotella timonensis* (p=0.03), *Megasphaera* (unclassified) (p=0.033,), and *Gardnerella vaginalis* (p=0.037)(D).
Data are represented as percentages in A, B and C as mean ± standard error of mean in D. The p-values by two-tailed unpaired t-test in C, and by two-tailed Welch's t-test in D. Dots in C and D depict individual patients. (n=87, ***$p < 0.001$; **$p < 0.01$; *$p < 0.05$.). Source data are supplied as a Source Data File.
.
KEY - CST I: Lactobacillus crispatus-dominant, CST III: Lactobacillus iners-dominant), CST IV: Lactobacillus spp. deplete, high diversity

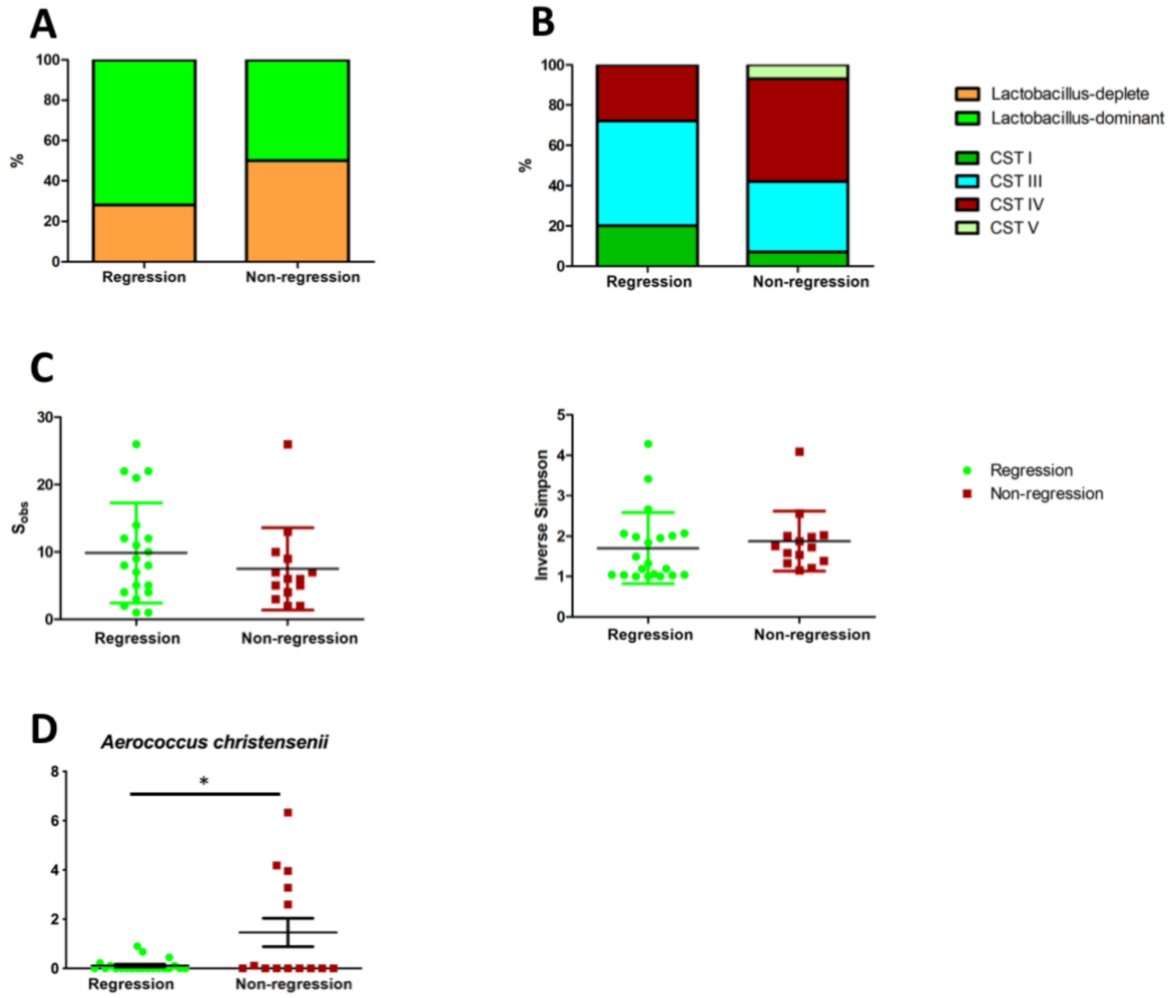

Supplementary Figure 3. Outcomes at 24 months according to VMB composition at 12 months in a subgroup of 35 women with ongoing disease

Women who had ongoing disease at 12 months follow-up were included in a further analysis to observe outcomes over the subsequent 12 months. There was a trend towards lower rates of regression in the presence of a Lactobacillus spp. deplete VMB (A) and CST IV (B), however this was not significant (B). There was also a trend towards higher species richness (species observed) in women who had not regressed at 12 months follow-up but this was not significant. Diversity (Inverse Simpson index) were not different between the two groups (C). The vaginal microbiota composition at the time of ongoing disease in the group of women who had not regressed a further 12 months later was characterised by an increased abundance of *Aerococcus christensenii* (p=0.037) (D).

Data are represented as percentages in A, B and C as mean ± standard error of mean in D. The p-values by two-tailed unpaired t-test in C, and by two-tailed Welch's t-test in D. Dots in C and D depict individual patients. (n=35, ***$p < 0.001$; **$p < 0.01$; *$p < 0.05$.). Source data are supplied as a Source Data File.

KEY - CST: Community state type, CST I: Lactobacillus crispatus-dominant, CST III: Lactobacillus iners-dominant), CST IV: Lactobacillus spp. deplete, high diversity

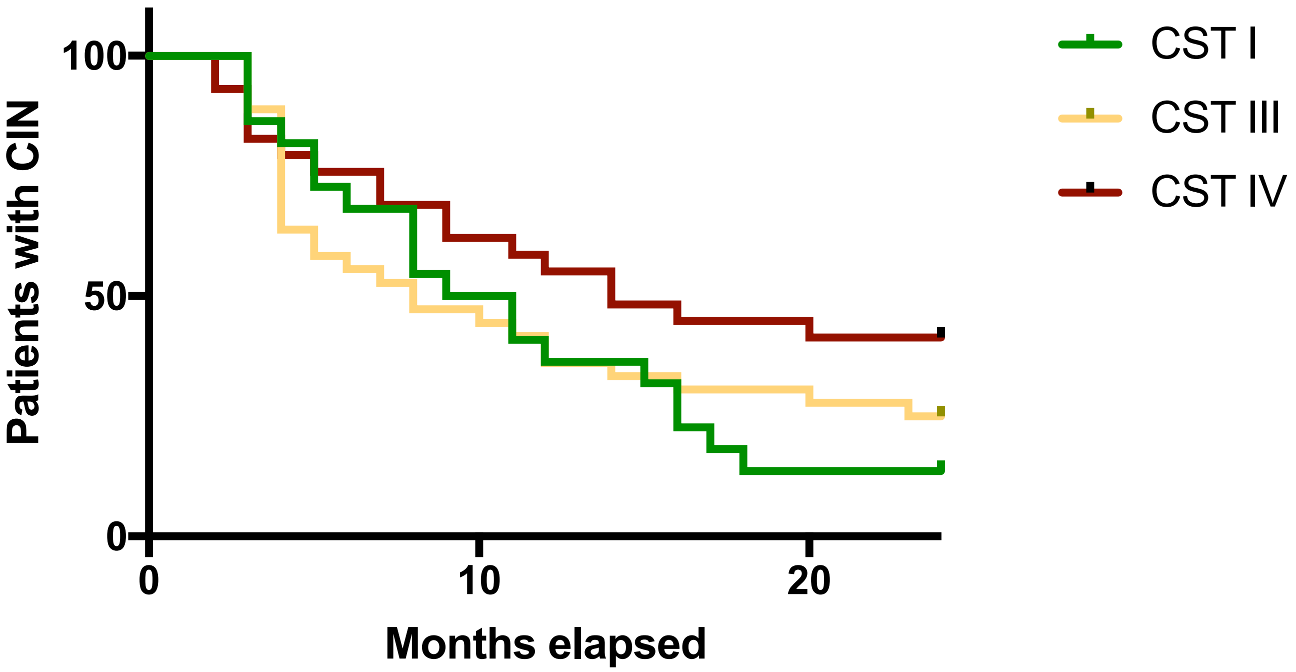

Supplementary Figure 4. Time to clearance of CIN according to VMB composition at species level
There was a trend towards slower disease regression in women with a CST IV at baseline compared to women with CST I or CST III at the time of CIN2 diagnosis (p-value=0.1864, Log-rank test). Source data are supplied as a Source Data File.
KEY - CST: Community state type, CST I: Lactobacillus crispatus-dominant, CST III: Lactobacillus iners-dominant), CST IV: Lactobacillus spp. deplete, high diversity, VMB: Vaginal microbiota

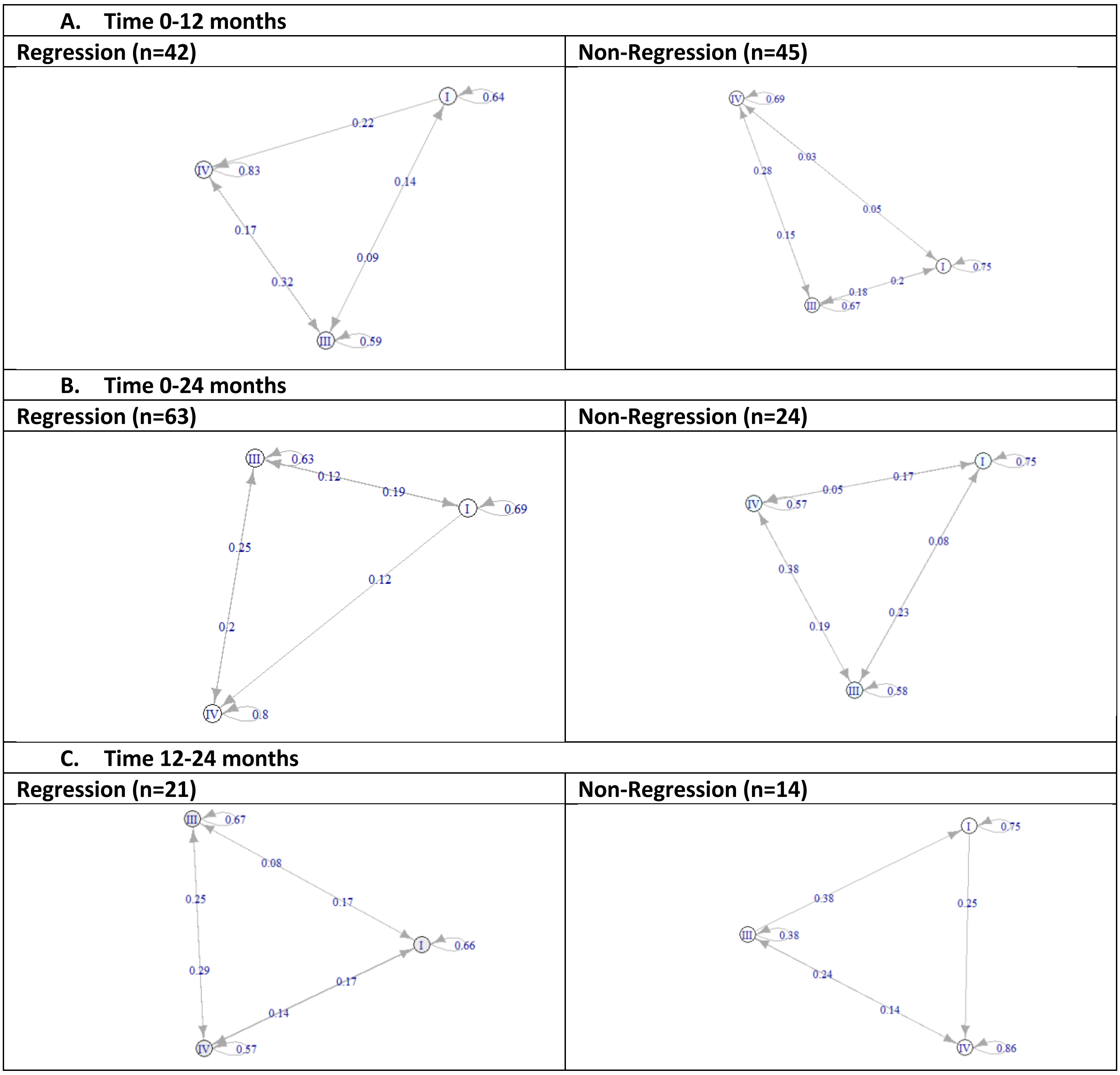

Supplementary Figure 5. Markov Modelling of transition probabilities of VMB composition between visits. CST IV was the most stable CST in regressors between 0-12 and 0-24 months, with CST I being most stable in non-regressors. The most frequent transitions were seen between CST IV to III in regressors at both timepoints, compared to CST III to IV in non-regressors. CST III was the most stable CST in regressors between 12-24 months, and CST IV was most stable in non-regressors.

Supplementary Table 1. Patient characteristics for 35 patients with persistent disease at 12 months included in subgroup analysis of outcomes at 24 months follow-up

	Non-regression at 24 months, n=14	Regression at 24 months, n=21	All, n=35
**Age, years**			p=0.8137
Mean (SD, range)	21.4 (2.5, 17.2 – 25.1)	21.2 (2.4, 17.3 – 25.9)	21.3 (2.4, 17.2 – 25.9)
**Ethnicity, n/N (%)**			p=0.8686
Caucasian	3/14 (21.4)	7/21 (33.3)	10/35 (28.6)
Black	5/14 (35.7)	6/21 (28.6)	11/35 (31.4)
Latin	4/14 (28.6)	6/21 (28.6)	10/35 (28.6)
Other ethnicity	2/14 (14.3)	2/21 (9.5)	4/35 (11.4)
**Smoking, n/N (%)**			p=0.3662
Current smoker	1/14 (7.1)	5/21 (23.8)	6/35 (17.1)
Non-smoker	13/14 (92.9)	16/21 (76.2)	29/35 (82.8)
**Weekly alcohol use, n/N (%)**			p=0.2588
Yes**, n/N (%)**	0/14 (0.0)	3/21 (14.3)	3/35 (8.6)
No	14/14 (100.0)	18/21 (85.7)	32/35 (91.4)
**Weekly drug use, n/N (%)**			p=0.2588
Yes	0/14 (0.0)	3/21 (14.3)	3/35 (8.6)
No	14/14 (100.0)	18/21 (85.7)	32/35 (91.4)
**Current/previous vaginal douching, n/N (%)**			p=0.6852
Yes	4/14 (28.6)	4/21 (19.0)	8/35 (22.9)
No	10/14 (71.4)	17/21 (81.0)	27/35 (77.1)
**Contraception, n/N (%)**			p=0.8534
Nil	3/14 (21.4)	4/21 (19.0)	7/35 (20.0)
Contraceptive injection	5/14 (35.7)	6/21 (28.6)	11/35 (31.4)
Other hormonal contraception	6/14 (42.9)	11/21 (52.4)	17/35 (48.6)
**Previous pregnancy, n/N (%)**			p=0.7026
Yes	8/14 (57.1)	13/21 (61.9)	21/35 (60.0)
No	6/14 (42.9)	8/21 (38.1)	14/35 (40.0)
**Number of sexual partners, n/N (%)**			p=0.5240
Mean (SD, Range)	10 (9, 2 – 36)	8 (8, 2 – 41)	9 (9, 2 – 41)
**Current sexual practice, n/N (%)**			p=0.6978
Abstinence	6/14 (42.9)	12/21 (57.1)	18/35 (51.5)
Monogamous	5/14 (35.7)	6/21 (28.6)	11/35 (31.4)
Non-monogamous	3/14 (21.4)	3/21 (14.3)	6/35 (17.1)
**History of anal intercourse, n/N (%)**			p=0.7210
Yes	4/14 (28.6)	8/21 (38.1)	12/35 (34.3)
No	10/14 (71.4)	13/21 (61.9)	23/35 (65.7)
**History of genital infections, n/N (%)**			p=0.7949
Chlamydia trachomatis	1/14 (7.1)	1/21 (4.8)	2/35 (5.7)
Neisseria gonorrhea	0/14 (0)	2/21 (9.5)	2/35 (5.7)
Trichomonas vaginalis	0/14 (0)	0/21 (0)	0/35 (0.0)
Genital warts	3/14 (21.4)	4/21 (19.0)	7/35 (20.0)
Syphilis	0/14 (0)	0/21 (0)	0/35 (0.0)
Bacterial vaginosis	3/14 (21.4)	3/21 (14.3)	6/35 (17.1)
Yeast infection	6/14 (42.9)	9/21 (42.9)	15/35 (42.9)
HSV	0/14 (0)	0/21 (0)	0/35 (0.0)
HIV	0/14 (0)	0/21 (0)	0/35 (0.0)
**HPV status, n/N (%)**			p=0.0259
Negative	7/14 (50.0)	3/21 (14.3)	10/35 (28.6)
Positive	6/14 (42.9)	17/21 (81.0)	23/35 (65.7)
High-risk HPV status			p=1.0000
Positive	5/6 (83.3)	13/17 (76.5)	18/23 (78.3)
Negative	1/6 (16.7)	4/17 (23.5)	5/23 (21.7)
HPV16 status			p=0.0987

Positive	3/5 (60.0)	2/13 (15.4)	5/18 (27.8)
Negative	2/5 (40.0)	11/13 (84.6)	13/18 (72.2)
HPV18 status			p=0.4902
Positive	1/5 (20.0)	1/13 (7.7)	2/18 (11.1)
Negative	4/5 (80.0)	12/13 (92.3)	16/18 (88.9)
Low-risk HPV status			p=0.6430
Positive	3/6 (50.0)	11/17 (64.7)	14/23 (60.9)
Negative	3/6 (50.0)	6/17 (35.3)	9/23 (39.1)
Number of HPV subtypes			p=0.1790
Single	1/6 (16.7)	9/17 (52.9)	10/23 (43.5)
Two or more	5/6 (83.3)	8/17 (47.1)	13/23 (56.5)
Unknown	1/14 (7.1)	1/21 (4.8)	2/35 (5.7)

The p-values represented are two-tailed Fisher's exact (if <5 observations in any group) and chi-square tests (where > 5 observations in every group).

KEY – HIV: Human immunodeficiency virus, HSV: Herpes simplex virus, HPV: Human papillomavirus, SD: Standard deviation

Supplementary Table 2. Diversity indices of total cohort for outcomes at 0-12 and 0-24 months.

Patient ID	CST	Genus	sobs	Inverse Simpson index	Clinical outcome 0-12 months	Clinical outcome 0-24 months
1008	4	Deplete	22	1.894423	Non-regression	Non-regression
2007	3	Dominant	8	1.037141	Non-regression	Regression
2014	3	Dominant	9	1.1327	Regression	Regression
2027	3	Dominant	3	1.127382	Non-regression	Non-regression
2035	4	Deplete	28	7.272846	Regression	Regression
2049	1	Dominant	2	1.011376	Regression	Regression
2058	1	Dominant	20	1.201932	Non-regression	Non-regression
2064	3	Dominant	10	1.36924	Regression	Regression
2069	3	Dominant	7	1.085423	Regression	Regression
2079	4	Deplete	22	1.846919	Non-regression	Regression
2091	1	Dominant	1	1	Regression	Regression
3002	3	Dominant	5	1.020644	Regression	Regression
3010	1	Dominant	3	1.048747	Non-regression	Non-regression
3020	4	Deplete	28	5.989802	Non-regression	Regression
3024	4	Deplete	11	2.149416	Non-regression	Non-regression
3030	4	Deplete	34	2.433875	Non-regression	Non-regression
3042	3	Dominant	6	1.585539	Non-regression	Non-regression
3047	4	Deplete	24	4.376825	Non-regression	Regression
3059	4	Deplete	15	3.55412	Non-regression	Regression
3060	3	Dominant	2	1.009091	Regression	Regression
3063	3	Dominant	5	1.441342	Regression	Regression
3067	4	Deplete	29	7.002309	Non-regression	Non-regression
3071	4	Deplete	6	2.839668	Non-regression	Non-regression
3084	4	Deplete	5	2.255596	Regression	Regression
3086	1	Dominant	12	2.62159	Regression	Regression
3088	3	Dominant	3	1.004538	Regression	Regression
5016	3	Dominant	4	1.060757	Regression	Regression
5018	4	Deplete	18	1.588333	Non-regression	Non-regression
5020	3	Dominant	7	1.049098	Non-regression	Regression
5059	4	Deplete	17	5.611873	Non-regression	Regression
5120	3	Dominant	7	1.317013	Non-regression	Regression
5123	1	Deplete	9	2.993274	Non-regression	Regression
5126	1	Dominant	1	1	Regression	Regression
5133	3	Dominant	6	1.620711	Non-regression	Non-regression
5137	4	Deplete	12	1.838749	Regression	Regression
5150	3	Dominant	5	1.932868	Regression	Regression
5156	3	Dominant	2	1.494898	Regression	Regression
5180	3	Dominant	5	1.884594	Regression	Regression
5196	3	Dominant	6	1.158813	Regression	Regression
5197	4	Deplete	13	3.316602	Non-regression	Non-regression
5204	3	Dominant	4	2.017016	Regression	Regression
6003	1	Dominant	2	1.025197	Regression	Regression
6006	3	Dominant	8	1.407329	Regression	Regression
7022	1	Dominant	3	1.009101	Regression	Regression
7037	3	Dominant	4	1.073257	Non-regression	Regression
7046	3	Dominant	13	1.125572	Non-regression	Non-regression
7049	4	Deplete	22	1.973324	Regression	Regression
7072	3	Dominant	4	1.102257	Regression	Regression
7077	3	Dominant	15	1.289564	Regression	Regression
8015	4	Deplete	12	1.36027	Non-regression	Non-regression
8016	1	Dominant	7	1.972965	Regression	Regression

8046	1	Dominant	2	1.002265	Regression	Regression
8060	3	Dominant	2	1.002265	Non-regression	Non-regression
10001	1	Dominant	3	1.006816	Regression	Regression
10007	3	Dominant	7	1.018332	Non-regression	Regression
10033	1	Dominant	3	1.022945	Non-regression	Regression
10053	3	Dominant	10	1.167229	Regression	Regression
10067	1	Dominant	5	1.194824	Non-regression	Regression
10074	4	Deplete	25	3.060152	Non-regression	Regression
10075	1	Dominant	4	1.013691	Regression	Regression
10081	4	Deplete	17	2.854255	Regression	Regression
10083	4	Deplete	11	1.150852	Non-regression	Non-regression
10085	3	Dominant	4	1.657886	Non-regression	Non-regression
10086	1	Dominant	2	1.011376	Non-regression	Regression
11005	4	Deplete	3	1.587212	Non-regression	Regression
11010	3	Dominant	3	1.036987	Non-regression	Regression
11027	4	Deplete	7	2.750283	Regression	Regression
11047	4	Deplete	17	1.832041	Non-regression	Regression
11067	4	Deplete	19	3.324325	Non-regression	Non-regression
11068	3	Dominant	7	1.441602	Regression	Regression
11076	1	Dominant	3	1.006816	Non-regression	Regression
11084	4	Deplete	18	4.405415	Non-regression	Non-regression
11086	1	Dominant	14	1.424514	Non-regression	Non-regression
11098	1	Dominant	3	1.041767	Regression	Regression
11107	3	Dominant	7	1.710078	Non-regression	Non-regression
11110	4	Deplete	8	1.103336	Regression	Regression
11118	4	Deplete	33	5.930412	Non-regression	Non-regression
11127	1	Dominant	3	1.004538	Non-regression	Regression
11134	1	Dominant	3	1.004538	Regression	Regression
11135	3	Dominant	8	1.246332	Regression	Regression
11136	3	Dominant	2	1.004535	Non-regression	Regression
11138	1	Dominant	7	1.030032	Regression	Regression
11139	4	Deplete	22	2.751657	Regression	Regression
11140	3	Dominant	3	1.341263	Non-regression	Non-regression
11141	4	Deplete	14	2.797809	Regression	Regression
20001	3	Dominant	9	1.0711	Non-regression	Non-regression
20004	3	Dominant	4	1.114759	Regression	Regression

KEY - CST: Community state type, Dominant: Lactobacillus-dominant, Dominant: Lactobacillus-deplete, sobs: species observed

Supplementary Table 3. Diversity indices of 35 patients included in subgroup analysis from 12-24 months

Patient ID	CST	Genus	sobs	Inverse Simpson index	Clinical outcome 12-24 months
1008	4	Deplete	26	4.087674	Non-regression
2007	3	Dominant	2	1.002265	Regression
2058	1	Dominant	3	1.149871	Non-regression
2079	4	Deplete	22	2.66055	Regression
3020	3	Dominant	1	1	Regression
3024	3	Dominant	11	1.056451	Regression
3030	4	Deplete	26	4.287452	Regression
3042	4	Deplete	2	1.585829	Non-regression
3047	3	Dominant	14	1.841389	Regression
3059	4	Deplete	10	1.759657	Non-regression
3067	3	Deplete	10	1.986149	Regression
3071	3	Dominant	2	1.985594	Non-regression
5018	4	Deplete	13	2.02747	Non-regression
5020	4	Deplete	9	2.009566	Regression
5059	3	Dominant	7	2.064821	Regression
5120	3	Dominant	4	1.188168	Regression
5123	1	Dominant	12	1.956868	Regression
5197	4	Deplete	7	1.529908	Non-regression
7037	4	Deplete	21	3.417585	Regression
8015	5	Dominant	5	1.728523	Non-regression
10007	4	Deplete	6	1.323833	Non-regression
10033	3	Dominant	12	1.321654	Regression
10067	1	Dominant	5	1.027632	Regression
10074	4	Deplete	22	2.071403	Regression
10083	3	Dominant	4	2.559996	Non-regression
10085	3	Dominant	7	1.209039	Non-regression
10086	3	Dominant	5	1.384359	Non-regression
11005	3	Dominant	8	1.039536	Regression
11010	4	Deplete	3	1.490103	Regression
11047	3	Dominant	5	1.186072	Regression
11067	3	Dominant	6	2.008121	Non-regression
11076	1	Dominant	8	1.037135	Regression
11107	4	Deplete	9	1.875449	Non-regression
11127	1	Dominant	4	1.039411	Regression
11136	3	Dominant	1	1	Regression

KEY - CST: Community state type, Dominant: Lactobacillus-dominant, Dominant: Lactobacillus-deplete

Supplementary Table 4. Vaginal microbiota composition at species and genus level according to HPV status and genotype at baseline.

	**HPV positive, n=79**	**hrHPV, n=73**	**HPV16, n=30**	**HPV 18, n=8**	**HPV negative, n=6**
**Lactobacillus-dominant, n=56**	52/79 (65.8)	49/73 (67.2)	20/30 (66.7)	4/8 (50.0)	4/6 (50.0)
***Lactobacillus* spp. depleted, n=29**	27/79 (34.2)	24/73 (32.8)	10/30 (33.3)	4/8 (50.0)	2/6 (33.3)
P value	>0.9999	>0.9999	>0.9999	0.6270	NA*
	**HPV positive, n=79**	**hrHPV, n=73**	**HPV16, n=30**	**HPV 18, n=8**	**HPV negative, n=6**
**CST I, n=21**	18/79 (24.1)	18/73 (24.7)	6/30 (20.0)	2/8 (25.0)	3/6 (50.0)
**CST III, n=35**	34/79 (43.0)	31/73 (42.5)	14/30 (46.7)	2/8 (25.0)	1/6 (16.7)
**CST IV, n=29**	27/79 (32.9)	24/73 (32.8)	10/30 (33.3)	4/8 (50.0)	2/6 (33.3)
P value	0.2705	0.3245	0.2369	0.6271	NA*

CST I (*Lactobacillus crispatus-dominant*), **CST III** (*Lactobacillus iners-dominant*), **CST IV** (*Lactobacillus* spp. deplete, high diversity)
CST: Community state type, hrHPV: high-risk Human Papilloma Virus,
χ^2 test compared to HPV -ve population. *no p-value because HPV negative population serve as control.
NB. Baseline HPV status unknown for 2 patients in cohort.
