# Supplementary material for: The vaginal microbiota associates with the regression of untreated cervical intraepithelial neoplasia 2 lesions
Source: Nat Commun. 2020 Apr 24;11:1999. doi: 10.1038/s41467-020-15856-y (PMC7181700; doi:10.1038/s41467-020-15856-y)
Supplement: Supplementary file 3 — Reporting Summary [file 41467_2020_15856_MOESM3_ESM.pdf]

nature research

Corresponding author(s): Maria Kyrgiou

Last updated by author(s): Mar 10, 2020

Reporting Summary

Nature Research wishes to improve the reproducibility of the work that we publish. This form provides structure for consistency and transparency in reporting. For further information on Nature Research policies, see Authors & Referees and the Editorial Policy Checklist .

Statistics

For all statistical analyses, confirm that the following items are present in the figure legend, table legend, main text, or Methods section.

n/a	Confirmed	
☐	☒	The exact sample size (*n*) for each experimental group/condition, given as a discrete number and unit of measurement
☐	☒	A statement on whether measurements were taken from distinct samples or whether the same sample was measured repeatedly
☐	☒	The statistical test(s) used AND whether they are one- or two-sided<br>*Only common tests should be described solely by name; describe more complex techniques in the Methods section.*
☐	☒	A description of all covariates tested
☐	☒	A description of any assumptions or corrections, such as tests of normality and adjustment for multiple comparisons
☐	☒	A full description of the statistical parameters including central tendency (e.g. means) or other basic estimates (e.g. regression coefficient) AND variation (e.g. standard deviation) or associated estimates of uncertainty (e.g. confidence intervals)
☐	☒	For null hypothesis testing, the test statistic (e.g. *F*, *t*, *r*) with confidence intervals, effect sizes, degrees of freedom and *P* value noted<br>*Give P values as exact values whenever suitable.*
☐	☒	For Bayesian analysis, information on the choice of priors and Markov chain Monte Carlo settings
☐	☒	For hierarchical and complex designs, identification of the appropriate level for tests and full reporting of outcomes
☒	☐	Estimates of effect sizes (e.g. Cohen's *d*, Pearson's *r*), indicating how they were calculated

Our web collection on statistics for biologists contains articles on many of the points above.

Software and code

Policy information about availability of computer code

Data collection	No software was used
Data analysis	Software used :<br>R (R Development Core Team 2008)(using custom code to create Markov models, available at https://github.com/anitamitra/Markov/tree/V1.0 and supplied separately as per Code and Software Submission Checklist & Vegan package - published protocol),<br>Mothur (using published pipeline as per manuscript),<br>STAMP (v2.1.3),<br>GraphPad Prism (v.8.0.1),<br>STATA(v.14),<br>USEARCH (v.11)

For manuscripts utilizing custom algorithms or software that are central to the research but not yet described in published literature, software must be made available to editors/reviewers. We strongly encourage code deposition in a community repository (e.g. GitHub). See the Nature Research guidelines for submitting code & software for further information.

Data

Policy information about availability of data

All manuscripts must include a data availability statement. This statement should provide the following information, where applicable:
- Accession codes, unique identifiers, or web links for publicly available datasets
- A list of figures that have associated raw data
- A description of any restrictions on data availability

Sequence data that support the findings of this study have been deposited in the European Nucleotide Archive's (ENA) Sequence Read Archive (SRA) repository; https://www.ncbi.nlm.nih.gov/sra with the accession code PRJEB31832. Basic metadata relating to disease outcome is available in the Supplementary Material to use alongside this to maintain anonimity. Further metadata is available upon request, however at the time of recruitment we did not seek explicit permission to

openly release all clinical data in a data repository. The source data underlying Figures 4 & 6 and Supplementary Figures 2, 3 & 4 are provided as a Source Data file.

Field-specific reporting

Please select the one below that is the best fit for your research. If you are not sure, read the appropriate sections before making your selection.

- [x] Life sciences
- [ ] Behavioural & social sciences
- [ ] Ecological, evolutionary & environmental sciences

For a reference copy of the document with all sections, see nature.com/documents/nr-reporting-summary-flat.pdf

Life sciences study design

All studies must disclose on these points even when the disclosure is negative.

Sample size	There was no sample size calculation performed, as due to the novelty of the study this was not possible. The samples are taken from a highly individual cohort of women with untreated CIN2 that is unlikely to ever be replicated. The original study was to determine the proportion on women who would regress, and at the time no sample size calculation was possible due to the novelty of the research question.
Data exclusions	No Data were excluded from the analysis
Replication	The findings cannot be reproduced. This is a highly individual cohort that to our knowledge has never been replicated.
Randomization	This is not relevant to our study. It is an observational study where all patients started with the same clinical disease state and all underwent a period of conservative management.
Blinding	There was no blinding because there was only one study group. All participants were treated in the same manner.<br>In addition there was no blinding for data analysis because this was not possible and would have no impact on the findings because the categorisation of disease, outcomes and vaginal microbiota were strictly defined.

Reporting for specific materials, systems and methods

We require information from authors about some types of materials, experimental systems and methods used in many studies. Here, indicate whether each material, system or method listed is relevant to your study. If you are not sure if a list item applies to your research, read the appropriate section before selecting a response.

Materials & experimental systems

n/a	Involved in the study	
x		Antibodies
x		Eukaryotic cell lines
x		Palaeontology
x		Animals and other organisms
	x	Human research participants
x		Clinical data

Methods

n/a	Involved in the study	
x		ChIP-seq
x		Flow cytometry
x		MRI-based neuroimaging

Human research participants

Policy information about studies involving human research participants

Population characteristics	Women between the ages of 16 to 26 years of age with histologically proven CIN2 were included
Recruitment	All adolescents aged 13-24 years who had abnormal cervical cytologic screening while attending one of the 12 participating clinics within Kaiser Permanente Northern California (KPNC) between 2002-2007 were eligible for recruitment. Referral for abnormal cytology included high-grade squamous intraepithelial lesions (HSIL), low-grade squamous intraepithelial lesions (LSIL), and atypical squamous cells-undetermined significance (ASC-US)/high-risk HPV positive, ASC-H (cannot exclude HSIL), or repeated ASC-US.<br>All patients subsequently found to have histologically proven CIN2 were subsequently included. We therefore believe that this would therefore eliminate any bias towards any kind of patient group or outcome as all grades of abnormality were considered for initial inclusion. Histological classification of cervical biopsies at baseline was performed by the local histopathologist and sent to the centralised laboratory for verification by a second histopathologist.
Ethics oversight	Ethical approval was obtained from the Institutional Review Boards of the University of California, San Francisco, and Kaiser Permanente, Northern California. At the time of recruitment we did not seek explicit permission to openly release all clinical data in a data repository but we stated to the research group that we would maintain their confidentiality and anonymity. We have therefore uploaded only the basic clinical data (regression/persistence and HPV status (given that this was performed as a research test and not part of their routine clinical management)) alongside sequence data in order to ensure that patients remain unidentifiable. Further data is available upon request from interested parties.

Note that full information on the approval of the study protocol must also be provided in the manuscript.
